# HIV cause-specific deaths, mortality, risk factors, and the combined influence of HAART and late diagnosis in Zhejiang, China, 2006–2013

**DOI:** 10.1038/srep42366

**Published:** 2017-02-15

**Authors:** Lin Chen, Xiaohong Pan, Qiaoqin Ma, Jiezhe Yang, Yun Xu, Jinlei Zheng, Hui Wang, Xin Zhou, Tingting Jiang, Jun Jiang, Lin He, Jianmin Jiang

**Affiliations:** 1 Zhejiang Provincial Center for Diseases Control and Prevention, 3399 Binsheng road, Binjiang District, Hangzhou, Zhejiang, China

## Abstract

To examine patterns of human immunodeficiency virus (HIV) cause-specific deaths, risk factors, and the effect of interactions on mortality, we conducted a retrospective cohort study in Zhejiang, China, from 2006 to 2013. All data were downloaded from the acquired immune deficiency syndrome (AIDS) Prevention and Control Information System. The Cox proportional hazards model was used to assess predictors of cause-specific death. The relative excess risk due to interaction and ratio of hazard ratios (RHR) were calculated for correlations between HAART, late diagnosis, and age. A total of 13,812 HIV/AIDS patients were enrolled with 31,553 person-years (PY) of follow-up. The leading causes of death of HIV patients were accidental death and suicide (21.5%), and the leading cause of death for those with AIDS was AIDS-defining disease (76.4%). Both additive and multiplicative scale correlations were found between receiving HAART and late diagnosis, with RERI of 5.624 (95% CI: 1.766–9.482) and RHR of 2.024 (95% CI: 1.167–2.882). The effects of HAART on AIDS-related mortalities were affected by late diagnosis. Early detection of HIV infection and increased uptake of HAART are important for greater benefits in terms of lives saved.

It has been estimated that by the end of 2011, there was a cumulative total of 78,000 people living with HIV (PLWH) in China, with 14,293 in Zhejiang province alone^1^. Since the beginning of the human immunodeficiency virus (HIV) epidemic, HIV infection has become the leading cause of infectious disease deaths in China^2^.

The first case of HIV infection in Zhejiang province was reported in 1985, with escalating numbers of cases over the subsequent three decades^3^. By the end of 2009, there was a cumulative total of 5,119 HIV cases in Zhejiang, of whom 1,261 (24.6%) had developed acquired immune deficiency syndrome (AIDS) and 533 (10.4%) who had died^3^. With a population of 66 million in Zhejiang province, the HIV prevalence rate among those 15 years and older was estimated at 0.03%. Sex between men was one of the major routes of transmission, and men who have sex with men (MSM) were the population with the highest prevalence of HIV in Zhejiang^3^,^4^.

After the initiation of China’s national Free Antiretroviral Treatment Program in 2002, Zhejiang province launched its program in 2003 and scaled up this program over time. Over 90% of HIV/AIDS patients needing treatment accepted highly active anti-retroviral therapy (HAART) by December 2010^5^,^6^.

Consequently, with the life expectancy of HIV patients substantially improved, causes of death among HIV-infected individuals have become more diverse, and deaths from AIDS-related and non-AIDS-related diseases have continued to change over the years^7^,^8^. Previous studies have documented factors that influence mortality among HIV-infected people including HAART treatment, late diagnosis, poverty, and little or no education^9^,^10^. Factors related to non-AIDS-associated deaths, however, have not been as extensively researched. To our knowledge, although the independent roles of HAART treatment and late diagnosis have been reported^11^,^12^, the interaction between them has not been addressed.

Our aim was to provide a broad and up-to-date report on risk factors and all causes of AIDS-related and non-AIDS-related mortality in Zhejiang province. We also evaluated whether there is an interaction between HAART treatment and late diagnosis on the risk of AIDS-related mortality.

## Methods

### Study design and data collection

This study is a retrospective observational cohort study in Zhejiang province with continued enrollment of HIV-infected persons at least 15 years old who were diagnosed between 2006 and 2013.

Data on PLWH were collected from the AIDS Prevention and Control Information System, which generates the national epidemiology database. The data in this system are collected compulsorily during routine tracking and clinical treatment of patients. The forms used for data collection are standardized nationwide, and include demographic, treatment, death, and laboratory information completed by health care facilities [hospital and local Centers for Disease Control and Prevention (CDC)] every 3 months for AIDS patients and every 6 months for HIV+ patients.

**Figure 1:**
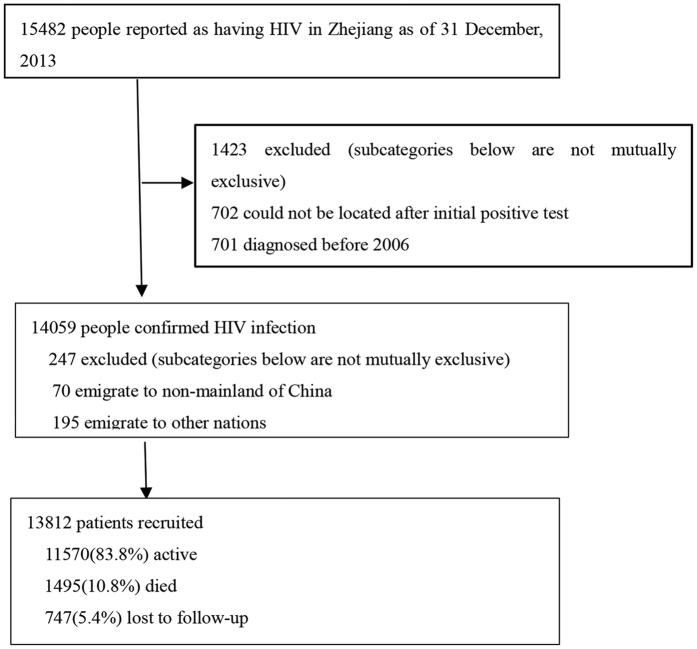
Study design flow chart for the human immunodeficiency virus/acquired immune deficiency syndrome (HIV/AIDS) cohort study, 2006–2013.

All the data from January 1, 2006 to December 31, 2013 were downloaded on July 1, 2014. Figure 1 shows our subject selection procedure. Patients who were not from mainland China or could not be located by the health care facility after diagnosis were excluded.

An individual was defined as eligible for this study when his/her HIV infection was confirmed by Western blotting. Date censoring occurred at: a) the date of the last visit for those lost to follow-up for at least half a year; b) the date of death (AIDS-related or non-AIDS-related); and c) the end of this study on December 31, 2013 for patients who continued to be followed up.

All records in the databases underwent quality control by regular database verification with regards to diagnosis of HIV infection, AIDS progression, and cause of death.

### AIDS and causes of death

Cases of AIDS were reported by the local CDC based on laboratory evidence of HIV infection and CD4+ T-cell count <200 cells/mm^3^ or at least one AIDS-defining illness as defined by the World Health Organization (WHO) stage 3 or 4 disease^13^. Mortality data were extracted from death certificates or by declaration of friends, parents, or relatives. Cause of death was specified in accordance with the 10th revision of the International Classification of Diseases. AIDS-associated death was defined as an AIDS-defining illness in accordance with WHO stage 3 or 4 disease. Non-AIDS-related deaths included all other known causes of death.

### Other key variables

Individuals were eligible to start HAART if they had a CD4+ T-cell count of <200 cells/mm^3^ or after 2011, <350 cells/mm^3^ (the most recent WHO recommendation for HAART^14^), or who were in WHO clinical stage 3 or 4. Individuals diagnosed with AIDS in the first year after initial examination were classified as a late diagnosis by calculating the time between the date of HIV diagnosis and AIDS diagnosis at <365 days.

### Ethics statement

Because patients’ data were collected from the National Center for AIDS/sexually transmitted disease (STD) Control and Prevention (NCAIDS) reporting system and analyzed anonymously, informed consent was not necessary. In China, we need to complete all of the work designated by the China CDC for HIV prevention and control, including HIV testing, HIV infection report, CD4 testing at least once a year, HAART, routine follow-up, death report, and so on. At the same time, we collect related data and upload it online as China CDC has requested. All of these procedures are routine work. This study is based on these data from routine work. We did obtain oral informed consent before HIV testing and CD4 testing as diagnosed.

### Statistical analysis

Statistical analyses were performed using SPSS statistical software (version 19.0, IBM, Armonk, NY, USA), and STATA (version 12.0, StataCorp LP, College Station, TX, USA). χ^2^ or Fisher’s exact tests were used to compare categorical variables. Mortality rates were calculated with a life table in person-years (PY); the numerator represents the number of deaths and the denominator represents the total person-years of patients at risk of death. Predictors for mortality were assessed by univariate and multivariate Cox proportional hazards regression. Crude and adjusted hazard ratios (HR) with 95% confidence intervals (95% CI) were estimated. The likelihood ratio test was used to evaluate model fit. To determine reasons for AIDS-related death and non-AIDS-related death caused by disease, those who died from drug overdose, suicide, or accidents were excluded from the model.

Potential additive and multiplicative interactions were also assessed for initiation of HAART and age and initiation of HAART and late diagnosis. In the additive interaction analysis, factors were recorded in which the stratum with the lowest risk was considered as the reference, while both factors were considered jointly. Relative excess risk due to interaction (RERI) and 95% CIs were used to evaluate additive interactions. If 95% CIs of RERI included 0, there was no obvious additive interaction. Multiplicative interactions were calculated by the ratio of hazard ratios (RHR) and 95% CIs. If 95% CIs of RHR included 1, there was no obvious multiplicative interaction. For unbiased HR for interaction, we adjusted for potential confounding factors including age, gender, education level, area of registry, area of residence, transmission route, last CD4+ T-cell count, and coinfection with tuberculosis (TB).

Significance was determined at the *p* ≤ 0.05 level; all hypothesis tests were two-sided.

## Results

### Deaths in Zhejiang province, 2006–2013

Enrollment and follow-up of the 13,812 HIV/AIDS patients in the registry with 31,553 PY follow-up is shown schematically in Fig. 1. The median age was 36.3 years (IQR 26.1–43.3); 10,682 (77.3%) were male; and 10,356 (75.0%) were residents of Zhejiang province. Of all individuals, 12,830 (92.9%) were infected through sexual activity, with 8,436 (61.1%) self-reporting as heterosexual and 4,394 (31.8%) self-reporting as homosexual.

Of the total 13,812 HIV/AIDS patients, 1,495 (10.5%) died between 2006 and 2013, with a yearly mean mortality rate of 4.1 (1,495/36,147) per 100 PY. Of the total 5,656 AIDS patients, 741 (13.1%) died, with a yearly mean mortality rate of 4.9 (741/15,166) per 100 PY.

**Figure 2:**
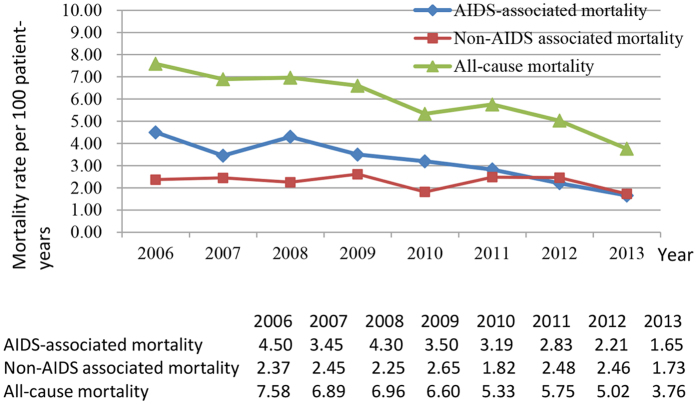
Mortality rate among HIV/AIDS patients in 2006–2013.

**Figure 3:**
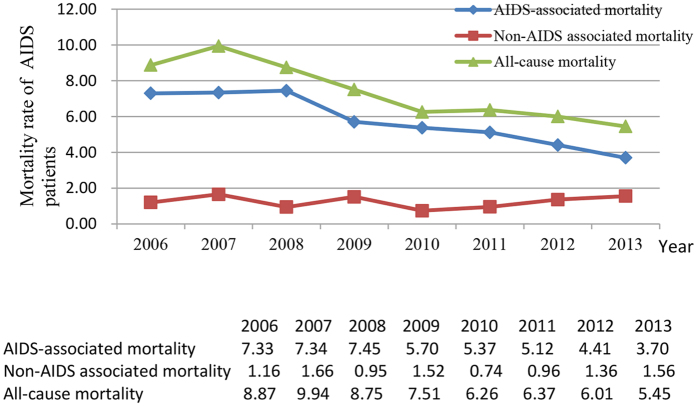
Mortality rate among AIDS patients in 2006–2013.

**Figure 4:**
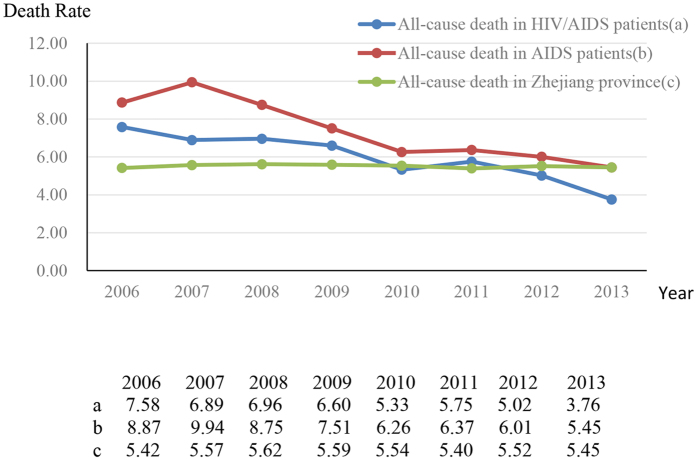
Death rates in HIV/AIDS patients, AIDS patients, and the population in Zhejiang province, 2006–2013.

**Figure 5:**
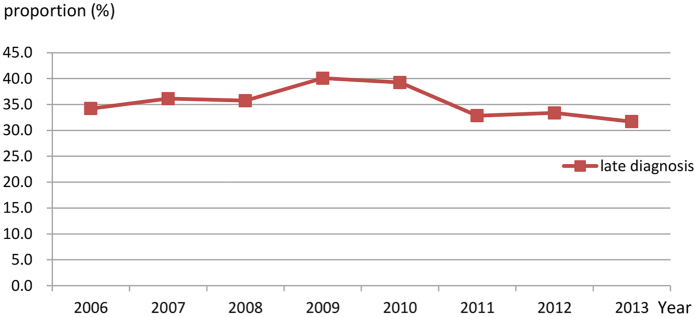
The proportion of late diagnosis among HIV/AIDS in 2006–2013.

Between 2006 and 2013, all causes of mortality decreased from 7.58 per 100 PY (2006) to 3.76 per 100 PY (2013) among HIV/AIDS patients (reduced by 50.5%), and from 8.87 per 100 PY (2006) to 5.45 per 100 PY (2013) among AIDS patients (reduced by 38.6%) (Figs 2 and 3). AIDS-associated mortality was 4.50 per 100 PY (2006) among HIV/AIDS patients and 7.33 per 100 PY (2006) among AIDS patients, and decreased to 1.65 per 100 PY (2013) (63.3%) and 3.70 per 100 PY (2013) (49.5%), reduced by 63.3% and 49.5%, respectively (Figs 2 and 3). Non-AIDS-associated mortality decreased from 2.37 per 100 PY (2006) to 1.73 per 100 PY (2013) among HIV/AIDS patients, but increased from 1.16 per 100 PY (2006) to 1.56 per 100 PY (2013) among AIDS patients (Figs 2 and 3). Compared with all causes of mortality in Zhejiang province, all causes of mortality in HIV/AIDS patients and in AIDS patients were much higher before 2010, but even lower after 2012 (Fig. 4). The proportion of late diagnosis among HIV/AIDS was decreased slightly, from 34.2% (2006) to 31.7% (2013) (Fig. 5).

### Cause of death by age and disease status

Of all deaths in our study, 64.9% (970/1495) were among AIDS patients, and 49.5% (260/525) and 60.2% (584/970) of deaths occurred at ages over 40 years in HIV patients and AIDS patients, respectively. Leading causes of death for HIV patients were accidental death and suicide (21.5%), non-AIDS-associated malignancies (17.5%), and lung disease (excluding cancer) (14.5%). For AIDS patients, the most frequent underlying causes of death were AIDS-associated death (76.4%) and non-AIDS-defining malignancies (4.3%). There were more suicides among HIV patients under 40 years old (Table 1). For all deaths, 49.6% died from AIDS-associated diseases, followed by suicide, accidental death, substance use (12.6%), non-AIDS-associated malignancies (9.0%), and lung disease (7.3%) (Table 1).

### Risk factors for AIDS-related and non-AIDS-related deaths

For AIDS patients, the three leading factors most strongly related to AIDS-associated death were having received HAART, compared to not having received HAART (HR 0.176, 95% CI 0.144–0.217), having been diagnosed with AIDS within one year of being diagnosed with HIV, compared to diagnosed with AIDS more than one year of HIV diagnosis (HR 4.260, 95% CI 3.344–5.426), and last CD4+ T-cell count (100–199 cells/mm^3^: HR 0.160, 95% CI 0.125–0.204; 200–499 cells/mm^3^: HR 0.059, 95% CI 0.045–0.079; ≥500 cells mm^3^: HR 0.034, 95% CI 0.017–0.068, compared to CD4+ T-cell count <100 cells/mm^3^) (Table 2). Other risk factors associated with AIDS-related mortality were age greater than 40 years, registry in Zhejiang province, resident of a different province, lower education, infection through heterosexual contact, and coinfection with TB (Table 2).

For all HIV/AIDS patients, the main risk factors related to non-AIDS-associated mortality were age greater than 60 years, compared to younger than 60 years (HR 5.755, 95% CI 3.814–8.683), late diagnosis, compared to early diagnoses (HR 2.277, 95% CI 1.618–3.205), infection by heterosexual contact, compared to infection by homosexual contact (HR 2.134, 95% CI 1.340–3.399), and last CD4+ T cell count (100–199 cells/mm^3^: HR 0.353, 95% CI 0.230–0.543; 200–499 cells/mm^3^: HR 0.234, 95% CI 0.164–0.334; ≥500 cells/mm^3^: HR 0.040, 95% CI 0.102–0.175, compared to CD4 + T-cell count <100 cells/mm^3^). HAART uptake (compared to without HAART, HR 0.141, 95% CI 0.102–0.196) was an important preventive factor (Table 2). In addition, being male, migrating to another province from Zhejiang, lower education, were also associated with non-AIDS-related mortality (Table 2).

### Combined influences of HAART and age and HAART and late diagnosis on AIDS-related mortality

The combined influences of HAART and age and HAART and late diagnosis on AIDS-related mortality are shown in Table 3. After adjusting for confounding factors, no interactions were observed between HAART treatment and age on an additive scale (RERI = 1.448; 95% *CI*:-0.088–2.983) and multiplicative scale (RHR = 0.999; 95%*CI*: 0.759–1.239). Both additive and multiplicative scale interactions were found between HAART and late diagnosis, with a RERI = 5.624 (95%*CI:* 1.766–9.482) and RHR = 2.024 (95%*CI*: 1.167–2.882).

## Discussion

In this seven-year study of mortality trends among HIV-infected patients in Zhejiang province, we found that all-cause mortality and AIDS-related mortality continued to decrease from 2006 to 2013. Other national cohort studies in China and Switzerland also reported substantial reductions in mortality of HIV/AIDS patients after HAART became available^6^,^15^. All-cause mortality and AIDS-related mortality both declined to the point that they were even lower than all-cause mortality in Zhejiang province and in China, but were still higher than that in some developed regions such as the United States and Europe^6^,^16^,^17^. Different statistical analyses and rates of HAART coverage should be taken into account. Even though the number of non-AIDS-related deaths increased markedly, our study showed a relative inequality in reduction between AIDS-related and non-AIDS-related mortality. A rapid decline was observed in AIDS-related mortality, whereas non-AIDS-related mortality decreased substantially and plateaued in 2010. The reductions in all-cause mortality, AIDS-associated mortality, and non-AIDS-related mortality represent a major public health success due to scaled-up treatment coverage, improvement of health care services, and early detection of HIV.

The increasing diversity of causes of death in the HAART era has been recognized^18^,^19^,^20^. In this study, patients with HIV continued to die of AIDS. Between 2006 and 2013, 76.4% of deaths among AIDS patients and 49.6% of deaths of HIV-infected people were due to AIDS. Several reports show that the proportion of deaths due to AIDS ranged from 10% to 74%^21^,^22^,^23^. We should consider that the distributions of causes of death in different studies are not directly comparable due to different characteristics of patients, such as gender, age groups, and socioeconomic status. However, it may also contribute to delayed diagnosis and earlier initiation of HAART^24^. Late diagnosis (HIV diagnosis within one year of diagnosis of AIDS) occurred in 26.4% of cases of HIV/AIDS patients in Zhejiang province in 2012^25^. People who do not know their HIV status may have a higher risk of death than diagnosed patients. Moreover, the differences in the definition of AIDS death and accuracy of AIDS diagnosis must be taken into account. To improve the quality of classification of causes of death, we adopted deep clearance of data to make key variables more accurate.

Even though AIDS-defining diseases are the leading cause of death in HIV/AIDS patients, they have decreased in favor of other causes. The leading causes of death of HIV patients were suicide (especially in young people), accidents, and substance use, accounting for 26.3%. This rate was lower than that reported in many cohorts, such as 32.7% in a population-based cohort study in Spain^18^. Several epidemiologic studies have shown that young people infected with HIV are more likely than older people to suffer from pressure from parents, friends, and society and react with suicide^26^,^27^. Further studies on suicide in HIV patients are necessary to identify specific risk factors.

Our findings are consistent with those already published that indicate that HAART and delayed diagnosis play an extremely important role in reducing mortality, both for AIDS-related and non-AIDS-related deaths after adjusting for potential confounding factors^28^,^29^,^30^,^31^. HAART provision to individuals in Zhejiang province started in 2004, with 23.9% coverage in 2006, scaled up to 67.2% in 2010, and peaked at 93.2% in 2013. With the improvement of hospitals providing HAART treatment, HIV-infected individuals have had better treatment services; e.g., accessibility to treatment and healthcare for disease complications. In our study, the adjusted hazard ratios of HAART for AIDS mortality and non-AIDS mortality were statistically significant, 0.070 and 0.138, respectively, indicating that HAART has a more powerful protective effect against AIDS mortality than non-AIDS mortality. Compared with HAART, the association between late diagnosis and mortality was much lower for both AIDS mortality and non-AIDS mortality.

The overall finding concurs with what is known about late diagnosis typically affecting mortality among HIV/AIDS patients. Late diagnosis of HIV-infected patients leads to late entry into HIV care, including HAART uptake. We analyzed the proportion of late diagnoses in 2006–2013 and found that it decreased slightly, accounting for almost 30%, which is lower than that in Brazil and in China^11^,^30^. The study in Brazil revealed that late entry into HIV care accounted for 95.5% of AIDS-related deaths in the first year after diagnosis; averting those deaths would have lowered the mortality rate by 39.5%^11^.

An important strength of our analysis is that it identified the combined influences of HAART treatment and late diagnosis and HAART treatment and age. The aim of enrollment of AIDS patients for interaction analyses was to diminish the impact of patients without need for HAART treatment. Based on our results, the impact of going without HAART and late diagnosis on the AIDS mortality rate is more additive than the two factors present individually. Patients who were older or diagnosed late were more likely to die and progress to AIDS than younger people and those diagnosed early^30^,^31^. However, no effect modification was observed between HAART and age on AIDS mortality, even though the association of age and mortality has been confirmed in several studies^32^,^33^. However, a study conducted in Spain revealed no differences in virology and CD4+ T-cell responses to HAART treatment for a delayed HIV diagnosis group^34^. To our knowledge, this is the first attempt to determine the possible correlation for mortality between HAART treatment and late diagnoses. The result revealed that it is effective to encourage patients to receive HAART treatment, especially those with low CD4+ T-cell counts. However, this open cohort study was conducted with short follow-up times, which may affect the measure modifications over a longer period of time. If the correlation is genuine, the underlying biological or statistical mechanisms merit further investigation.

As with all cohort studies, selection bias from loss to follow-up may exist. We compared the demographic characteristics of participants at follow-up and those lost to follow-up and found that most of the patients who were lost to follow-up were younger (80% were ≤40 years old) and female migrants. The differential selection bias may lead to overestimation of the associations. For AIDS-related mortality, only 1.5% of patients were lost to follow-up, and selection bias can be overlooked. In addition, even though potential confounding factors were considered on the basis of prior knowledge and confounding assessment, there were still some unknown and unassessed confounders generating bias. Despite potential limitations, the current study had a large sample size, and we were able to evaluate both of the main associations between HAART and other factors.

In conclusion, our study shows that the decline in mortality occurred stably in the HAART era. There is strong evidence that HAART treatment and early diagnosis have substantially reduced AIDS-related and non-AIDS-related mortality. The effect of HAART on AIDS mortality was affected by age and time of diagnosis, suggesting that detection of potential HIV infection and having HAART treatment (among individuals in need of treatment) are important and bring even larger benefits in terms of lives saved.

## Additional Information

**How to cite this article:** Chen, L. *et al*. HIV cause-specific deaths, mortality, risk factors, and the combined influence of HAART and late diagnosis in Zhejiang, China, 2006–2013. *Sci. Rep.* **7**, 42366; doi: 10.1038/srep42366 (2017).

**Publisher's note:** Springer Nature remains neutral with regard to jurisdictional claims in published maps and institutional affiliations.

## Figures and Tables

**Table 1:** Specific causes of death among HIV and AIDS patients by age in 2006–2013.

	Total	HIV patients			AIDS patients		
		<40-years	≥40-years	P	<40-years	≥40-years	P
No. of deceased participants (%)	1495 (100)	265 (50.5)	260 (49.5)	—	386 (39.8)	584 (60.2)	—
**AIDS-defining disease, total**	741 (49.5)	0 (0)	0 (0)	—	307 (79.6)	434 (74.3)	0.061
**Non-AIDS-defining malignancies**	134 (9.0)	27 (10.2)	65 (25.0)	0	8 (2.1)	34 (5.8)	0.005
Lung malignancies	18 (1.2)	4 (1.5)	10 (3.8)	0.111*	0 (0)	4 (0.7)	—
HCC	16 (1.1)	3 (1.1)	7 (2.7)	0.218*	0 (0)	6 (1.0)	—
Other	100 (6.7)	20 (7.5)	48 (18.5)	0	8 (2.1)	24 (4.1)	0.082
**Liver failure**	35 (2.3)	11 (4.2)	10 (3.8)	0.859	6 (1.6)	8 (1.4)	0.814
**Digestive system failure**	15 (1.0)	7 (2.6)	3 (1.2)	0.339*	1 (0.3)	4 (0.7)	0.654*
**Heart failure, total**	26 (1.7)	3 (1.1)	13 (5.0)	0	1 (0.3)	9 (1.5)	0.058*
**Central nervous system, total**	33 (2.2)	6 (2.3)	17 (6.5)	0.017*	3 (0.8)	7 (1.2)	0.748*
Stroke	17 (1.1)	3 (1.1)	9 (3.5)	0.035*	2 (0.5)	3 (0.5)	0.66*
Other	16 (1.1)	3 (1.1)	8 (6.9)	0.599*	1 (0.3)	4 (0.7)	0.654*
**Lung disease (excl. cancer)**	109 (7.3)	23 (8.7)	53 (20.4)	0	13 (3.4)	20 (3.4)	0.962
**Suicide**	66 (4.4)	27 (10.2)	13 (5.0)	0.025	10 (2.6)	16 (2.7)	0.888
**Accidental death**	89 (6.0)	46 (17.4)	27 (10.4)	0.021	6 (1.6)	10 (1.7)	0.850
**Substance use, total**	33 (2.2)	19 (7.2)	6 (2.3)	0.009	5 (1.3)	3 (0.5)	0.277*
Injecting drug use	31 (2.1)	19 (7.2)	4 (1.5)	0.002*	5 (1.3)	3 (0.5)	0.277*
Chronic alcohol use	2 (0.1)	0 (0.0)	2 (0.8)	—	0 (0)	0 (0)	—
**Other**	93 (6.2)	42 (15.8)	25 (9.6)	0.032	11 (2.8)	15 (2.6)	0.794
**Unknown**	121 (8.1)	54 (20.4)	28 (10.8)	0.002	15 (3.9)	24 (4.1)	0.862

Note: *Fisher’s exact test.

HIV: human immunodeficiency virus; AIDS: acquired immune deficiency syndrome.

**Table 2:** Demographics of AIDS-related and non-AIDS-related death and predictors between 2006 and 2013.

	AIDS-related death^a^			Non-AIDS-related death^b^		
	Mortality rate	HR (95% CI)	Adjusted HR (95% CI)	Mortality rate	HR (95% CI)	Adjusted HR (95% CI)
**Gender**						
Female	3.72 (150/4036.6)	1	1	1.69 (165/9766.9)	1	1
Male	5.31 (591/11129.6	1.295 (1.082–1.551)	0.973 (0.772–1.226)	2.23 (589/26389.7)	1.262 (1.003–1,586)	1.868 (1.264–2.761)
**Age at the end of follow-up**						
<40	3.82 (307/8029.8)	1	1	1.62 (356/21993.3)	1	1
40~59	5.12 (299/5834.5)	1.299 (1.108–1.524)	1.269 (1.029–1.565)	2.12 (252/11903.5)	1.907 (1.532–2.374)	1.908 (1.343–2.711)
≥60	10.37 (135/1301.9)	2.327 (1.900–2.851)	1.798 (1.368–2.362)	6.46 (146/2259.7)	6.972 (5.524–8.800)	5.755 (3.814–8.683)
**Area of registry**						
Zhejiang	5.21 (462/8864.5)	1	1	1.90 (351/18456.2)	1	1
Other provinces	4.43 (279/6301.7)	0.878 (0.757–1.109)	0.706 (0.517–0.965)	2.28 (403/17700.4)	0.761 (0.630–0.918)	0.640 (0.399–1.027)
**Area of residence**						
Zhejiang	4.42 (512/11574.0)	1	1	1.67 (427/25583.2)	1	1
Other province	6.37 (229/3592.2)	1.530 (1.309–1.789)	1.536 (1.108–2.129)	3.09 (327/10573.3)	1.453 (1.193–1.769)	1.763 (1.084–2.866)
**Education**						
Primary school or less	5.68 (620/10917.8)	1	1	2.52 (646/25675.4)	1	1
Junior high school	3.31 (83/2507.5)	0.581 (0.462–0.731)	0.740 (0.550–0.996)	1.27 (78/6117.7)	0.611 (0.464–0.804)	1.079 (0.719–1.619)
Senior high school or more	2.03 (35/1727.2)	0.344 (0.245–0.483)	1.030 (0.683–1.554)	0.70 (30/4281.4)	0.187 (0.110–0.319)	0.369 (0.147–0.926)
**Transmission routes**						
MSM	2.39 (91/3801.8)	1	1	0.80 (74/9200.4)	1	1
Heterosexual	5.65 (563/9962.4)	2.513 (2.014–3.137)	1.377 (1.066–1.777)	2.18 (480/21977.0)	4.673 (3.328–6.561)	2.134 (1.340–3.399)
IDU	4.44 (37/834.1)	2.351 (1.603–3.448)	1.017 (0.626–1.651)	3.87 (139/3592.7)	4.262 (2.605–6.973)	1.716 (0.831–3.544)
Other/unknown	8.81 (50/567.8)	4.444 (3.145–6.279)	1.180 (0.746–1.865)	4.40 (61/1386.4)	12.470 (7.841–19.830)	1.756 (0.756–4.080)
**Late diagnosis**						
No	2.16 (146/6771.1)	1	1	2.16 (586/27083.5)	1	1
Yes	7.09 (595/8395.1)	2.364 (1.963–2.846)	4.260 (3.344–5.426)	1.85 (168/9073.1)	0.981 (0.721–1.101)	2.277 (1.618–3.205)
**HAART**						
No	40.51 (496/1224.3)	1	1	2.94 (643/12681.1)	1	1
Yes	1.76 (245/13941.9)	0.055 (0.047–0.064)	0.176 (0.144–0.217)	1.22 (111/23475.4)	0.112 (0.087–0.144)	0.141 (0.102–0.196)
**TB coinfection**						
No	2.79 (376/13485.0)	1	1	1.07 (335/31449.2)	1	1
Yes	6.19 (81/1309.5)	2.234 (1.757–2.841)	1.554 (1.180–2.047)	0.90 (17/1896.4)	0.127 (0.656–1.934)	0.828 (0.455–1.505)
Unknown	76.4 (284/371.7)	18.165 (15.485–21.309)	2.833 (2.247–3.572)	14.30 (402/2811.0)	14.338 (11.821–17.390)	2.403 (1.622–3.562)
Last CD4+ T-cell count (cells/mm^3^)						
0–99	26.24 (343/1306.9)	1	1	4.41 (58/1313.9)	1	1
100–199	3.13 (89/2841.2)	0.125 (0.099–0.159)	0.160 (0.125–0.204)	1.26 (36/2862.3)	0.294 (0.194–0.446)	0.353 (0.230–0.543)
200–499	0.84 (73/8734.7)	0.036 (0.028–0.047)	0.059 (0.045–0.079)	0.57 (104/18327.7)	0.135 (0.098–0.186)	0.234 (0.164–0.334)
≥500	0.46 (10/2170.8)	0.019 (0.010–0.037)	0.034 (0.017–0.068)	0.26 (20/7821.5)	0.061 (0.037–0.102)	0.102 (0.059–0.175)

^a^Among AIDS patients.

^b^Among HIV/AIDS patients.

HR: hazard ratio; CI: confidence interval; MSM: men who have sex with men; IDU: Injected drug use; HAART: highly active antiretroviral treatment; TB: tuberculosis.

**Table 3:** Interaction of AIDS mortality risk between HAART and major risk factors among AIDS patients.

Variables	HAART	No. of case/person time (PY)	Crude HR (95% CI)	Adjusted HR (95% CI)^a^
Age				
≥40	No^c^	285/473.7	1.00	1.00
≥40	Yes	149/6662.7	0.050 (0.031–0.079)	0.172 (0.140–0.211)
<40	No	212/750.6	0.577 (0.482–0.690)	0.732 (0.601–0.891)
<40	Yes^b^	97/72779.1	0.018 (0.011–0.076)	0.141 (0.059–0.273)
Interaction	Additive	RERI = 1.448 (95%*CI*: −0.088–2.983)		
	Multiplicative	RHR = 0.999 (95%*CI*: 0.759–1.239)		
Late diagnosis				
Late	No^c^	402/402	1.00	1.00
Late	Yes	193/7993.4	0.038 (0.032–0.046)	0.075 (0.043–0.132)
Early	No	94/822.6	0.194 (0.152–0.247)	0.176 (0.129–0.230)
Early	Yes^b^	52/1245	0.015 (0.011–0.021)	0.026 (0.007–0.098)
Interaction	Additive	RERI = 5.624 (95%*CI:* 1.766–9.482)		
	Multiplicative	RHR = 2.024 (95%*CI*: 1.167–2.882)		

^a^Adjusted on age (<40 = 1, ≥40 = 0), gender (female = 1, male = 0), education background (primary or less = 1, junior high school = 2, senior high school or more = 3), area of registry (Zhejiang province = 1, other provinces = 0), area of residence (Zhejiang province = 1, other provinces = 0), transmission route (MSM = 1, heterosexual = 2, IDU = 3, other/unknown = 4), TB (No = 1, Yes = 2, Unknown = 3), CD4+ T-cell count (0–99 = 1; 100–199 = 2; 200–499 = 3; ≥500 = 4).

^b^The reference category for measure of interaction on an additive scale.

^c^The joint effects category for estimation of additive interaction.
